# Comparative Study on Automated Cell Nuclei Segmentation Methods for Cytology Pleural Effusion Images

**DOI:** 10.1155/2018/9240389

**Published:** 2018-09-12

**Authors:** Khin Yadanar Win, Somsak Choomchuay, Kazuhiko Hamamoto, Manasanan Raveesunthornkiat

**Affiliations:** ^1^Faculty of Engineering, King Mongkut's Institute of Technology Ladkrabang, Bangkok, Thailand; ^2^School of Information and Telecommunication Engineering, Tokai University, Tokyo, Japan; ^3^Department of Pathology, Faculty of Medicine, Srinakharinwirot University, Nakhon Nayok, Thailand

## Abstract

Automated cell nuclei segmentation is the most crucial step toward the implementation of a computer-aided diagnosis system for cancer cells. Studies on the automated analysis of cytology pleural effusion images are few because of the lack of reliable cell nuclei segmentation methods. Therefore, this paper presents a comparative study of twelve nuclei segmentation methods for cytology pleural effusion images. Each method involves three main steps: preprocessing, segmentation, and postprocessing. The preprocessing and segmentation stages help enhancing the image quality and extracting the nuclei regions from the rest of the image, respectively. The postprocessing stage helps in refining the segmented nuclei and removing false findings. The segmentation methods are quantitatively evaluated for 35 cytology images of pleural effusion by computing five performance metrics. The evaluation results show that the segmentation performances of the Otsu, k-means, mean shift, Chan–Vese, and graph cut methods are 94, 94, 95, 94, and 93%, respectively, with high abnormal nuclei detection rates. The average computational times per image are 1.08, 36.62, 50.18, 330, and 44.03 seconds, respectively. The findings of this study will be useful for current and potential future studies on cytology images of pleural effusion.

## 1. Introduction

Globally, cancer is one of the deadliest diseases with high morbidity and mortality. In 2015, approximately 14 million new cases were diagnosed, and over 8 million deaths were estimated worldwide [1]. Ferlay et al. [2] estimated that the death toll due to cancer is set to rise dramatically by approximately 70% in the coming decades. Fortunately, the mortality and morbidity associated with cancer can be reduced, with a high potential for cure if cancer is diagnosed and treated at an early stage.

**Figure 1:**
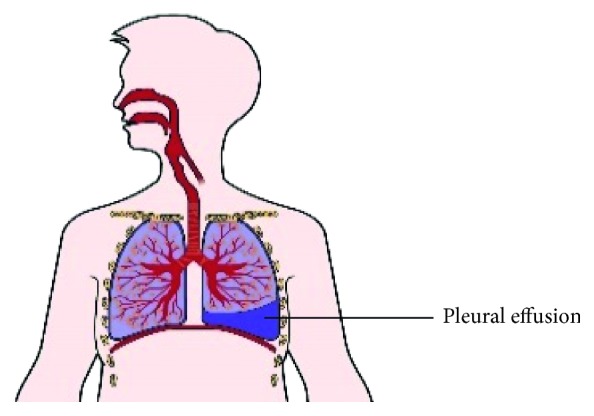
Excessive amount of pleural effusion.

When cancer grows or flows in the pleura cavity between the lungs and the chest wall, it causes a malignant pleural effusion, which is the excessive collection of pleural fluid, as shown in Figure 1 [3].

Fifty percent of cancer patients have a high possibility of developing malignant pleural effusion. Both primary and metastasis cancers can be diagnosed from pleural effusion [4]. The most frequently occurring primary cancer in pleural effusion is mesothelioma. The most common types of metastasis cancers are the cancers of the breasts, lungs, ovaries, and blood, including other unknown primary sites.

Pleural effusion can be detected using several imaging approaches such as X-ray, ultrasound, computed tomography (CT), and magnetic resonance imaging (MRI), including other tests such as urine and blood tests. For the assessment of malignancy, a cytological examination is performed by pathologists because it is simple, cheap, less invasive, and highly effective.

The cytological exam is a manual procedure wherein cytologists or experts visually investigate every single cell on the cytology glass slides using a microscopic camera, identify any abnormality in a cell, and finally make a decision. However, the procedure is time-consuming and requires good skill; moreover, it is tedious and prone to inter- and intraobserver variations. In addition, the diagnosis accuracy strongly depends on the attention and expertise of cytologists. These factors have inspired us to implement a computer-aided diagnosis (CAD) system. The CAD system can help relieve the workload on cytologists, accelerate the diagnosis procedure, eliminate the inter- and intraobserver variations in the diagnosis, and describe the quantitative results, thus complementing and enhancing the opinions of the cytologists. To implement a CAD system for cancer cells, cell nuclei segmentation is a prerequisite because cancer cells are largely observed on the basis of the morphological changes in the cell nuclei. Therefore, it is crucial to select an accurate and effective cell nuclei segmentation method that can help precisely delineate the nuclei contours.

## 2. Literature Review

Several promising nuclei segmentation approaches have been proposed for different types of microscopic images, for example, cervical and breast cells. Cell nuclei segmentation methods for breast cell images [5–10] and cervical cell images [11–15] have been reported. Moreover, methods have been proposed to segment cell nuclei on prostate cancer cell images [16, 17] and microscopic blood cell images [18, 19]. Significant efforts have been made to segment the cell nuclei from other types of images such as bone marrow images, lung sputum cells images, brain cell images, liver and thyroid images, bladder and skin tissue images, and brain glioma images in various studies [20–24], respectively.

Although many robust and effective methods for nuclei segmentation have been reported [5–24], they are designed to address specific types of cell images such as those of the breast, cervical, blood, and prostate. In different types of cell images, the structure of the cells and their corresponding gray-level distributions vary significantly. Therefore, the aforementioned methods cannot be directly employed to segment all types of images on the basis of the diversity of the images.

Only a few studies have detected malignant cells from cytology pleural effusion images. Zhang et al. [25] reported a method to detect malignant cells from pleural effusion images using integrated fuzzy edge detection and Otsu's method. Chen et al. [26] presented a method on the basis of the wavelet and morphology transform to detect malignant cells from pleural effusion images.

However, a preprocessing stage for removing noise and enhancing contrast is not considered in these methods, thus reducing the accuracy of the detection system. In addition, the methods are not focused on the segmentation process and there is a lack of quantitative evaluation of the proposed methods. To address the shortcomings, we recently reported two cell nuclei segmentation methods [27, 28]. However, nuclei segmentation in cytology pleural effusion images is still a challenging task because of the high cell population, their varieties, overlapping cells, and poor cell contrast. There is scope to further improve the cell segmentation method for cytology pleural effusion images. Thus, more observations are required to implement and determine the most feasible segmentation method.

In the cytology and histology image analysis, nuclei segmentation often revolves around thresholding techniques, clustering techniques, and active contour techniques. Thresholding techniques are quite simple ones in nuclei segmentation. Every pixel in the image is determined into nuclei or background depending on the image intensity. In spite of providing the effective segmentation performance for the images with uniform background, they are sensitive to noises, uneven background, and intensity heterogeneity inside the images [17, 25, 29, 30, 31]. Clustering techniques are unsupervised methods that attempt to group the pixels having similar features into different objects without prelabeling the samples [10, 14, 19, 20]. Active contour techniques are based on moving the deformable splines inside the images to find nuclei contours using the gradient information [6, 16, 32, 33]. Graph cut methods recently have a great interest in nuclei segmentation and yield the good segmentation performance [9, 11]. Other popular techniques are variants of watershed method and concavity analysis method which are frequently used for isolating the overlapping nuclei that is critical issue of cytology and histology image segmentation. The above stated techniques are widely utilized in either individual or integrating together in many microscopy image analyses, especially in cervical cytology and breast histology image segmentation. According to their simplicity, reproducibility and affordable cost of processing methods are (still) considered in our new designs of cytology pleural effusion analysis. We emphasize on simple and classical image segmentation methods, that is, thresholding, clustering, active contour, and graph cut methods, to extract the nuclei region from the background in cytological pleural effusion images.

In this paper, we experimentally employed twelve cell nuclei segmentation methods individually from four techniques: (i) thresholding techniques, (ii) clustering techniques, (iii) graph cut techniques, and (iv) active contours. Each method involves three main stages: preprocessing, segmentation, and postprocessing. In the preprocessing stage, contrast-limited adaptive histogram equalization (CLAHE) and median filtering are used to enhance the image quality and remove the small noises. In the segmentation stage, we proposed twelve segmentation methods, including (1) Otsu's method, (2) Isodata thresholding method, (3) maximum entropy thresholding method, (4) cross entropy thresholding, (5) minimum error thresholding, (6) fuzzy entropy thresholding method, (7) adaptive thresholding method, (8) k-means clustering, (9) fuzzy c-means clustering, (10) mean shift clustering, (11) Chan–Vese level set, and (12) graph cut methods. Finally, the boundaries of the segmented cell nuclei are refined, and false findings are eliminated using morphological methods. The methods are quantitatively evaluated in terms of five performance metrics, and their accuracies are later compared. Finally, a discussion along with the advantages and disadvantages of the methods is presented.

The examined methods aim to extract the nuclei regions from the surrounding objects and background in the images. These methods attempt to segment out all possible nuclei regions regardless of single laying or overlapping. They are not capable of separating the overlapping cells. It should also be noted that the performance metrics measured here are aimed to compute the correctness of the segmented pixels by matching with the pixels in the hand-drawn ground truth image. When computing the nuclei detection rate, each connected region from the segmentation results is considered as one nucleus regardless of the number of nucleus inside the region. The overlapping issue is not taken into account in the evaluation processes of segmentation accuracy and nuclei detection rate. Nevertheless, the undersegmentation errors of the overlapping cells can affect the final decision of CAD system. Thus, the isolation of overlapping nuclei and extraction of interregional walls will be remained as future study. On the bright side, the examined methods have potential to integrate with overlapped splitting methods to split the overlapped nuclei.

The rest of this paper is structured as follows.  Section 3 presents the datasets and segmentation methods.  Section 4 presents a benchmark of the experimental results, including parameters tuning and discussion. Finally, the conclusions of this study are presented in Section 5.

## 3. Materials and Methods

### 3.1. Dataset Description and Ground Truth Segmentation

**Figure 2:**
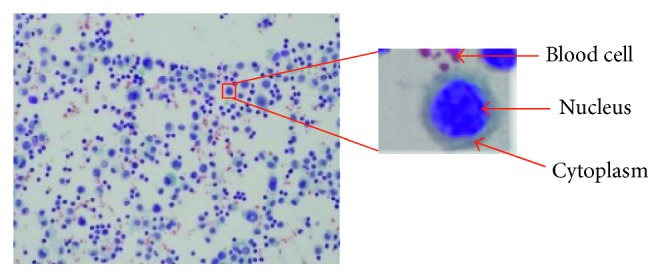
Pap smear images of pleural effusion and its components.

We are not aware of any publicly available dataset for cytology pleural effusion images. Thus, we prepared a local dataset with the help of a hospital. The studied dataset is based on cytology glass slides of pleural effusion specimens obtained with the cooperation of experts from the Department of Pathology, Faculty of Medicine, Srinakharinwirot University, Thailand. The samples were taken from the pleural space using thoracentesis procedure, spread on the glass slides, and stained using classical Papanicolaou (Pap) staining method. The images were captured using an Olympic microscope mounted on the digital camera. The dataset comprises 35 images of cytology pleural effusion containing healthy cells, benign cells, and cancerous cells. The resolutions of the images were 4050 × 2050 pixels, stored in 8-bit RGB space.  Figure 2 shows the sample and component of the cytology pleural effusion image. To set the gold standard, the ground truth images were prepared with the help of experts from the hospital. First, computer vision researchers manually delineated the cell nuclei. The experts then verified and annotated the cell nuclei and pathology cells.

### 3.2. Cell Nuclei Segmentation Framework

**Figure 3:**
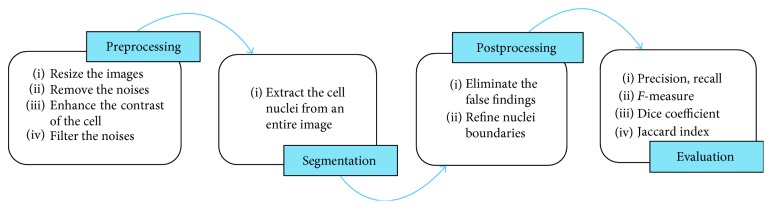
Generalized framework of cell nuclei segmentation for cytology pleural effusion images.

Three main stages are considered to automatically segment the cell nuclei.  Figure 3 shows the segmentation framework. In the next section, we introduce the details of each stage.

### 3.3. Preprocessing Stage

**Figure 4:**
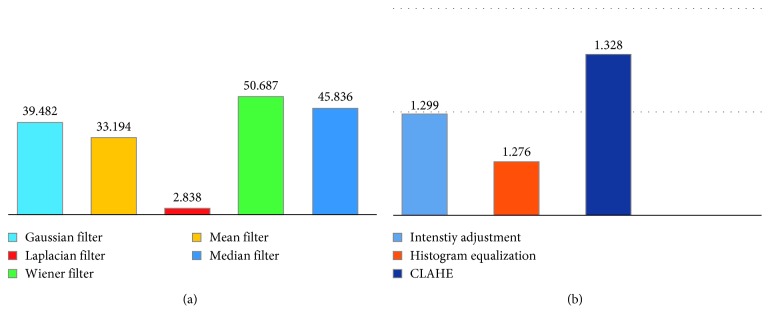
Image quality assessment metrics: (a) comparison of filtering methods in terms of peak signal-to-noise ratio (PSNR) and (b) comparison of different contrast enhancement methods in terms of the contrast improvement index (CII).

**Figure 5:**
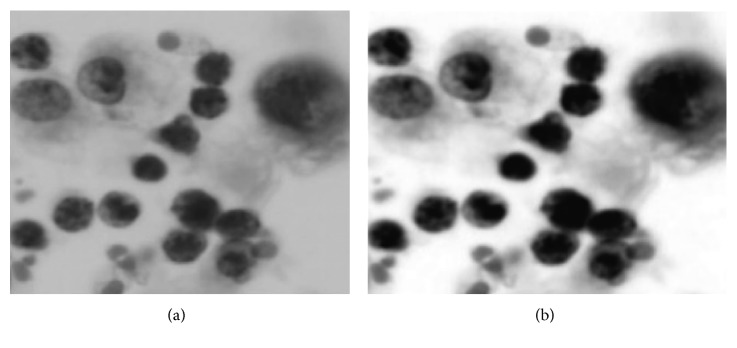
Preprocessing stage: (a) grayscale image and (b) preprocessed image after median filter and CLAHE (note that the image was cropped for better visibility).

The preprocessing stage is an essential step in improving the quality of the image. First, to reduce the computational load, the original input image is resized to resolutions of 1052 × 1052 pixels. The resized image is then converted into different color spaces using the segmentation methods. The cytology images might contain debris, noises, or stained artefacts because of the uneven illumination or dirt on the camera surface resulting from the image acquisition process. Moreover, many images are poor in terms of contrast. Therefore, it is required to suppress the noises and artefacts and enhance the cell contrast. First, to denoise the image, we employed five filtering methods, namely, Gaussian filter, Laplacian filter, Wiener filter, median filter, and mean filter. The peak signal-to-noise ratio (PSNR) for each method is computed. The PSNR is used to assess the quality of the filtered image. The higher the PSNR, the better is the image quality.  Figure 4(a) compares the results of the PSNR. We selected the median filter because it exhibits the highest PSNR. The median filter is nonlinear method to suppress the noises by windowing the noisy image. Default window size 3 × 3 is used to remove the small noises. To enhance the cell contrast, three enhancement methods, namely, histogram equalization, intensity adjustment, and CLAHE, are applied. The contrast improvement index (CII) is computed for each method. The CII is utilized for assessing the performance of the image enhancement techniques in terms of the luminance, contrast, and structure. The higher the CII value, the better is the contrast.  Figure 4(b) compares the results of the CII. The CLAHE is selected because it results in the highest CII. CLAHE with 8-bit histogram bins is utilized to enhance the contrast of the cell.  Figure 5 shows the resulting image after the CLAHE and median filter are applied.

### 3.4. Segmentation Stage

The segmentation step is an important step toward automatic image analysis. This step aims to discriminate between the foreground (the desired object) and the background of the image. The objective of this stage is to extract the cell nuclei from the entire image. In this section, we briefly summarize the twelve segmentation methods. We categorized them into four groups: thresholding, clustering, active contour, and graph-based techniques.

#### 3.4.1. Thresholding Techniques

The thresholding technique is the simplest segmentation method in terms of the gray-level image histogram. It aims to discriminate the foreground and the background by selecting an adequate threshold value. The threshold value can be global or local. A single optimal threshold is utilized for the whole image in the global thresholding, whereas the threshold for each pixel is computed depending on its local properties in the local approach. Many of thresholding methods take the normalized histogram of the image as the input parameter.

*(1) Otsu's Thresholding Method*. Otsu's method, which is invented by Nobuyuki Otsu, is one of the global thresholding methods. The aim of Otsu's method is to determine the optimal threshold that minimizes the intraclass variance [34]. The algorithm steps are given in Algorithm 1.

*(2) Isodata Thresholding Method*. The Isodata thresholding method is one of the global image thresholding methods wherein the following iterative procedure is employed [35]. It requires the initial threshold value as the input. The mean intensity of the image histogram is set as the initial value. The processing steps of Isodata thresholding are given in Algorithm 2.

*(3) Maximum Entropy Thresholding*. The maximum entropy thresholding method is one of the global thresholding methods. Similar to Otsu's method, an optimal threshold is selected in the maximum entropy thresholding method by maximizing the information measured between the object and the background [36]. It takes the normalized histogram of the image as the input parameter. The processing steps are summarized in Algorithm 3.

*(4) Cross Entropy Thresholding*. The cross entropy thresholding is one of the entropy methods. Numerous algorithms have been developed for the cross entropy thresholding. Here, we focus on the one proposed by Li and Lee, which is summarized as follows [37]. Similar to maximum entropy thresholding, cross entropy thresholding takes the histogram of the image as the input parameter.  Algorithm 4 describes the processing steps of cross entropy thresholding.

*(5) Fuzzy Entropy Thresholding*. Fuzzy entropy is defined as the measure of uncertainty of a fuzzy set, the procedure of which is given below. It requires two input parameters: (i) histogram of the image to compute the probability distribution and (ii) fuzzy membership function as given in [38]. The algorithm steps are summarized in Algorithm 5.

*(6) Minimum Error Thresholding*. In this method, the image segmentation is based on the average pixel optimization [39]. It requires the normalized histogram of the gray-level image as the input parameter. The idea behind this thresholding technique is presented in Algorithm 6.

*(7) Adaptive Thresholding*. Adaptive thresholding is the most famous local thresholding method for images with uneven illumination. It aims to select threshold values for each region based on its local properties. The local window size is empirically set as 12. We summarized the adaptive thresholding procedures as in Algorithm 7 [40]:

#### 3.4.2. Clustering Techniques

Clustering-based segmentation methods aim to group the collection of pixels into clusters. The pixels in the same cluster are closely related to one another.

*(1) K-Means Clustering*. The k-means clustering is one of the clustering methods wherein the data are divided into a specific number of groups by minimizing the within-class variance [41]. The processing steps of k-means clustering based segmentation is presented in Algorithm 8.

*(2) Fuzzy C-Means Clustering*. The fuzzy c-means clustering is one the most popular fuzzy clustering methods, wherein the data are partitioned into two or more fuzzy clusters by maximizing the objective function [42].  Algorithm 9 summarizes the steps involved in this technique.

*(3) Mean Shift Clustering*. Among the clustering-based segmentation methods, the mean shift segmentation is known as an advanced and highly useful technique. In the mean shift, a window is defined for each data point and the mean is subsequently computed. The center of the window is shifted to the mean and the iteration is performed until it converges [43].  Algorithm 10 describes the processing steps of mean shift clustering-based segmentation technique.

#### 3.4.3. Graph-Based Segmentation Technique

Graph-based models consider the image as a weighted graph. Every pixel in the image is considered as the node in the graph. The similarities between two nodes are stated as edge weights.

*(1) Min Cut*. A graph cut is a partition of the graph directly or indirectly into two disjoint subsets. The graph is partitioned into clusters using the min cut method. Each cluster is considered as an image segment. The min cut method uses the highly connected subgraph (HCS) algorithm to find the clusters [44]. It can be formulated as follows:

(1) $$\begin{array}{c} \text{cut}\left(X,Y\right)=\sum w\left(i,j\right), \end{array}$$

where *i* ∈ *X*, *j* ∈ *Y*, and *X* and *Y* are two partitioned disjoint sets.

#### 3.4.4. Active Contour Segmentation Technique

Active contour models (or snakes) aim to delineate the objects using the energy minimization function. It is performed by assigning the object boundary as the initial contour and subsequently evolving the contour to detect the desired object boundary by driving image forces [45].

*(1) Active Contour without Edges (Chan–Vese)*. Among many active contour methods, the active contour without edges, known as the Chan–Vese method, is widely used in cell segmentation. It helps detect the objects without a gradient. It has the ability to segment smoothed contour objects by shrinking the contours and works well on convex objects.

### 3.5. Postprocessing Stage

Postprocessing is an important step to optimize the segmentation results. While most of the segmented regions obtained through the segmentation step will likely correspond to the nuclei regions, there may also be the existence of false findings such as blood cells and artefacts, which must be filtered out. Therefore, it is essential to remove those spurious regions and retain the valid nuclei. A series of the morphological operations is utilized to remedy above problems [46]. Firstly, the morphological filtering method is applied to remove the small objects since the artefacts and blood cells are usually smaller than the actual nuclei. The processing of eliminating the spurious objects is given as Pseudocode 1.

**Figure 6:**
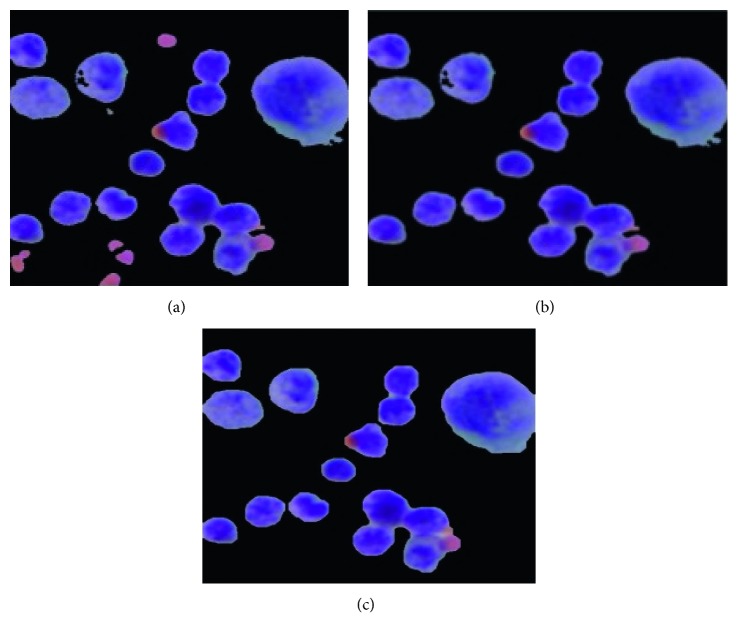
The sample visual results of before and after postprocessing stage: (a) candidate nuclei extracted using OTSU thresholding, (b) after removing spurious objects using morphological filtering, and (c) after refining the contours of the nuclei using morphological opening and closing.

As described in the above pseudocode, it is required to specify the size of *P* which is the threshold between the actual nuclei and the spurious regions. The optimal value of *P* is empirically set as 1500 pixels. After removing false findings, we further applied the morphological closing and opening operations for nuclei shape's refinement and simplification. The structuring element (SE) with disk shape and radius (*R*)is used. *R* is set as 5 and 12 for opening and closing, respectively. Equations (2) and (3) formulate opening and closing operations, respectively:

(2) $$\begin{array}{c} {\text{actual}}_{\text{nuclei}}\cdot \text{SE}=\left({\text{actual}}_{\text{nuclei}}\;⊖\text{ SE}\right)\;⊕\text{ SE}, \end{array}$$

(3) $$\begin{array}{c} {\text{opened}}_{\text{nuclei}}\cdot \text{SE}=\left({\text{opened}}_{\text{nuclei}}\;⊕\text{ SE}\right)\;⊖\text{ SE}, \end{array}$$

where ⊕and⊖ represent the dilation and erosion, respectively. The sampled visual results of before and after postprocessing are depicted in Figure 6.

## 4. Benchmark Setting

### 4.1. Experimental Results

**Figure 7:**
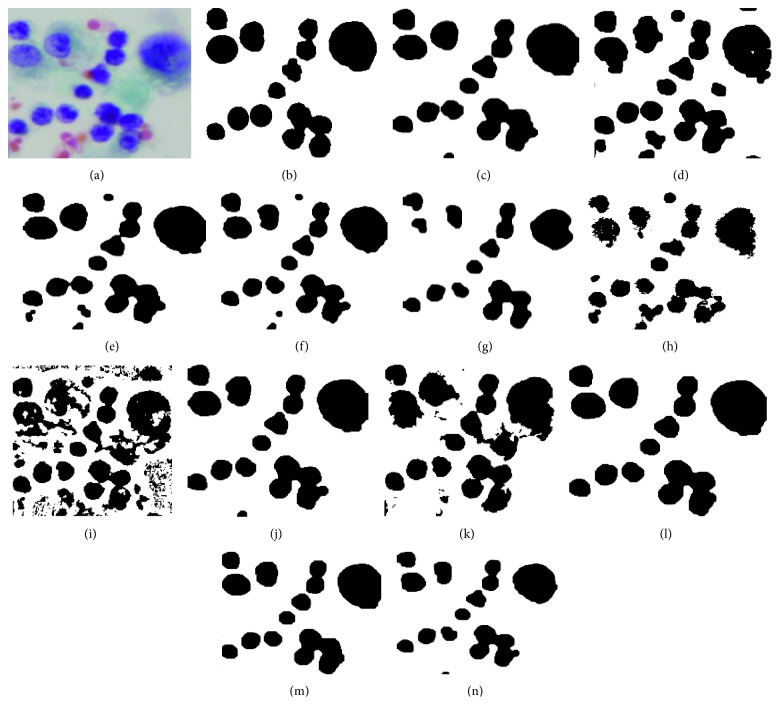
Visual cell nuclei segmented using twelve segmentation methods: (a) original image, (b) ground truth image, (c) Otsu's method, (d) Isodata, (e) maximum entropy, (f) cross entropy, (g) minimum error, (h) fuzzy entropy, (i) adaptive thresholding, (j) k-means clustering, (k) fuzzy c-means clustering, (l) mean shift clustering, (m) Chan–Vese method, and (n) graph-based min cut (note that the images were cropped for better visibility here).

This study was carried out using MATLAB (2013 version) on a computer with an Intel Core i7 processor clocked at 2.50 GHz and with 8 GB of RAM. A local dataset of 35 cytology images of pleural effusion and its ground truth images are used. In this study, we considered three main stages to extract the cell nuclei. The first stage is used to deal with the image quality. We employed different enhancement and filtering methods. The image quality assessment metrics, namely, CII and PSNR, are computed to select the best ones. Based on the CII and PSNR results, we selected CLAHE and median filtering methods to enhance the image quality. The segmentation stage is the most important stage in extracting the cell nuclei regions. We experimentally employed twelve segmentation methods, as explained in Section 3. Finally, the preprocessing stage is performed to refine the boundaries of the segmented cell nuclei and remove the undesired regions using morphological operations. As the segmentation stage is paramount, the segmentation results vary depending on the segmentation methods.  Figure 7 shows the visual results of the cell nuclei segmented using different segmentation methods.

To quantitatively evaluate the segmentation methods, five pixel-based performance metrics, namely, precision, recall, measure, Jaccard Index (JI), and Dice similarity coefficient (DSC), were computed for each algorithm. The examined methods are evaluated by comparing with hand-drawn ground truth images. Each connected region in the segmented results is considered as one nucleus while ignoring the number of nucleus inside the region. Ground truth images are also prepared the same way.  Figure 7 depicts the sample of ground truth image. The performance measures can be formulated as follows:

(4) precisionPre=true  positivetrue  positive+false  positive,recallRe=true  positivetrue  positive+false  negative,FmeasureFm=2 ∗ precision ∗ recallprecision+recall,JI=true  positivetrue  positive+false  positive+false  negative,DSC=2∗true  positive2 ∗ true  positive+false  positive+false  negative.

**Figure 8:**
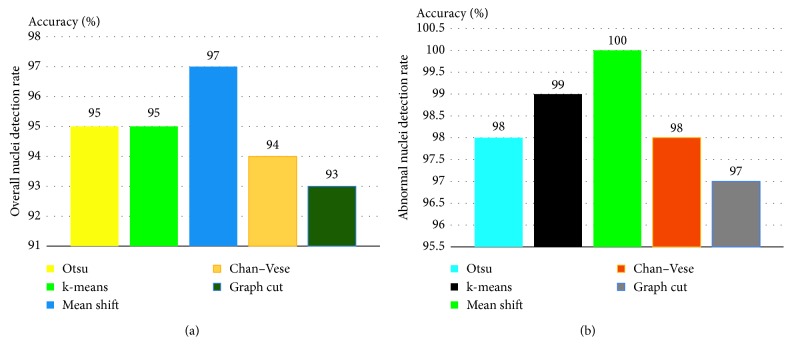
Comparison of nuclei detection rates in terms of the recall value: (a) overall nuclei detection rate and (b) abnormal cell nuclei detection rate.

**Figure 9:**
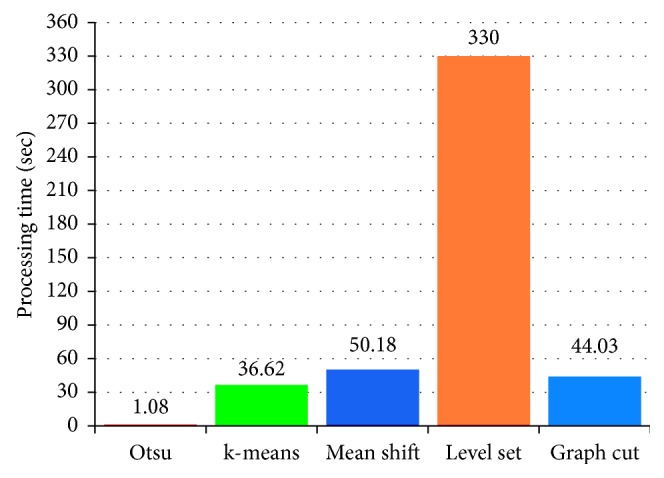
Processing time of five highlighted methods.

The performance metrics were used to quantitatively evaluate the cell nuclei in the segmented image individually.  Table 1 lists the evaluation results. The compared quantitative results show that the segmentation performances of Otsu's method, k-means, mean shift, Chan–Vese level set method, and graph-based min cut are excellent. The accuracies meet clinical requirements. For the highlighted methods, we further evaluated the nuclei detection rate (NDR) of the images depending on the recall value. The NDR is considered as true positive when the recall is greater than 60%. The overall NDR of each algorithm is computed and compared, as shown in Figure 8(a). Similar to the NDR, we estimated the abnormal NDR.  Figure 8(b) shows the comparison results. The comparison results show that the mean shift clustering method exhibits the best performance in terms of the overall NDR and abnormal NDR. To evaluate the time complexity of each method, the computational time of each method is computed and compared, as shown in Figure 9. Otsu's method is found to be relatively simple and fast. In contrast, the Chan–Vese method is computationally expensive.

### 4.2. Parameters Tuning and Discussion

The highlighted segmentation methods are discussed along with their adjustable parameters, advantages, and limitations. The experiment results show that the performances of the segmentation methods strongly depend on the tuning parameters. Therefore, it is required to properly select the most relevant parameters for our applications. We experimentally set and adjusted different parameters in each segmentation method and selected the most effective one for all images.

As Otsu's method is a nonparametric method, it is not required to specially assign prior parameters. However, Otsu's method is sensitive to outliers. To deal with this issue, the CLAHE and median filter methods are employed to enhance the image quality before applying Otsu's method. The cytology pleural effusion images comprise three main parts: cell nuclei, cytoplasm and blood cells, and background. As the color of the nuclei region appears to be dark purple, with other parts appearing lighter in color, the image histogram is assumed to be a bimodal distribution. Thus, Otsu's method provides relatively good performance in our application. Otsu's method is relatively simple and the result is promising. Therefore, it can be applied to real applications. However, the performance is degraded when the image contains significant noises because the method is sensitive to noise.

**Figure 10:**
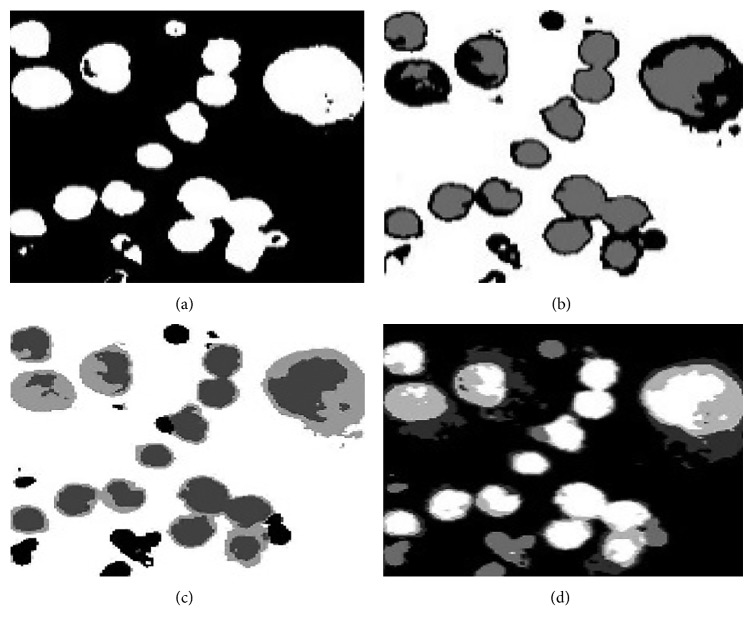
Image index labeled with different *k* clusters: (a) *k *= 2, (b) *k *= 3, (c) *k *= 4, and (d) *k *= 5.

The segmentation result of the k-means clustering method strongly depends on initializing the *k* clusters. A poor initialization can significantly affect the clustering performance and result in a poor convergence speed. Therefore, we set multiple *k* clusters for the test and chose the most effective one. The numbers of *k* clusters were set as 2, 3, 4, and 5. When *k* is 3, the nuclei are mixed with other image components. Thus, it was difficult to separate only the nuclei regions from the mixed clustered regions. When *k* is 4, 5, or more, the nuclei are broken into multisegmented images because of the high variation in the pixel intensity within the nuclei. Hence, the nuclei regions should be obtained from multiple images. When *k* is 2, the nuclei are segmented in a straightforward manner. In addition, the nuclei appear dark in color with some regions remaining bright. This fact also supports in setting up the value of *k* as 2 to cluster two groups (dark and bright colors).  Figure 10 shows the visual segmented cell nuclei with different *k* clusters. Moreover, it is worth noticing that the k-means clustering method performs well for round-shaped objects. Hence, the method is effective in segmenting cell nuclei because the cell nuclei are largely round shaped. In addition, it is simple, fast, and easy to implement. However, the disadvantage is that the k-means clustering is extremely sensitive to the *k* clusters and performs badly when the clusters are convex shaped.

**Figure 11:**
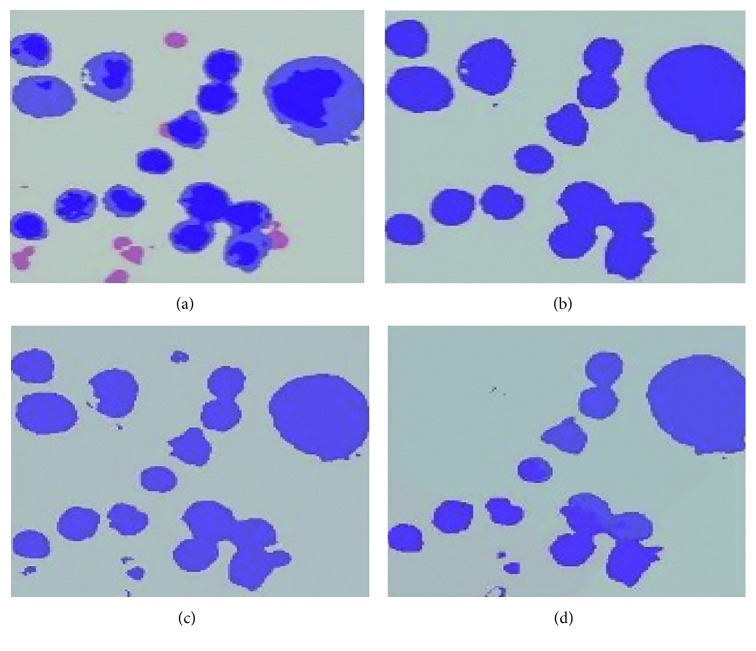
Different clustering results in terms of different bandwidth (bw) sizes: (a) bw = 0.1, (b) bw = 0.2, (c) bw = 0.3, and (d) bw = 0.4.

In contrast to the k-means clustering method, the mean shift is a nonparametric clustering method. It is not necessary to define the clusters and restrict the cluster shape. Nevertheless, the clustering result of the mean shift strongly depends on the bandwidth size. It is required to carefully select the most relevant size for particular applications. In our experiments, we experimentally set multiple bandwidth sizes and chose the best one.  Figure 11 shows the significant differences in the segmentations in terms of the bandwidth sizes. The experiment results show that a bandwidth size of 0.2 exhibits the best clustering performance, appropriate for cell nuclei segmentation. The advantage of the mean shift is that it is not necessary to initialize the cluster numbers; moreover, the method need not be robust to outliers. Only the size of the bandwidth is required to be set. The limitation of the mean shift is that it is computationally expensive compared to other clustering methods, as many windows need to be shifted, thus making many computations redundant.

**Figure 12:**
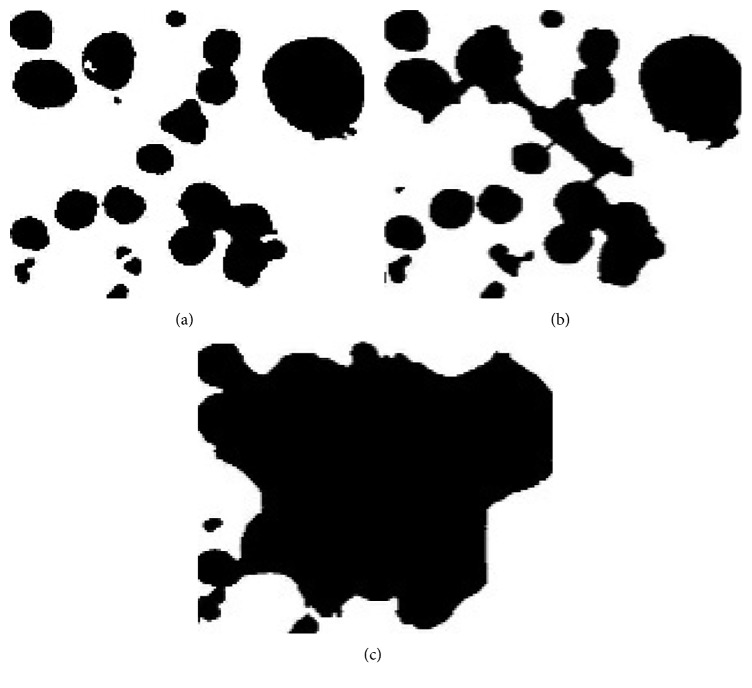
Segmentation results obtained using different iterations: (a) iterations = 500, (b) iterations = 300, and (c) iterations = 100 (note that iterations were tested on cropped region of image for better comparison, not on the real input images).

In the Chan–Vese level set method, the boundaries of the regions are used as a mask, which is initial contour evolution of the segmentation start. To achieve a fast and accurate output, we initially specified the mask that is close to the nuclei regions. The mask either shrinks or expands based on the image features. In addition, it is crucial to specify an appropriate maximum iteration for the contour evolution. The iteration is stopped if the maximum iteration is reached, when the energy remains constant, or when the contour is not moving. The maximum number of iterations affects the largest variation in the segmentation results. Therefore, we experimentally tested with different iterations and chose the most effective one for all images. However, it is the main limitation, as the images contain various types of cells. It is difficult to fix the number of iterations if more images are added into the datasets. If a large number of maximum iterations are set, the computation becomes expensive. In contrast, a small number of iterations lead to undersegmentation, because the iteration is stopped before finishing the contour evolution.  Figure 12 depicts the segmentation results obtained though different iteration numbers.

**Figure 13:**
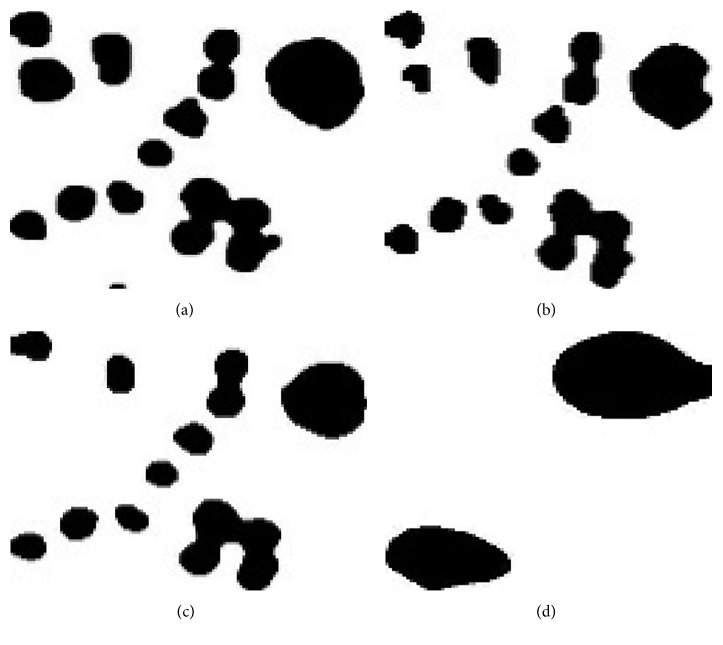
Variation in segmentation results in terms of different alpha values (av): (a) av = 0.3, (b) av = 0.5, (c) av = 10, and (d) av = 15.

In the graph-based min cut segmentation, two parameters need to be defined. The first one is alpha, which is the penalty parameter with respect to the total variation term. For the case wherein the image-edge weights are incorporated, alpha is given by the constant in all cases. For the case with image-edge weights, alpha is given using the two pixel-wise weighted functions. The second parameter is the step size of the augmented Lagrangian method, the optimal range of which is (0.3, 3). We set it as 0.3, as it is not significantly different for segmentation. A significant variation in the segmentation result is found when setting up different values of alpha. We experimentally tested with different alpha values and chose the best one.  Figure 13 depicts the visual result of the segmentation in terms of the alpha values. We chose 0.3 as the alpha value in our application. The graph-based min cut method is simple, easy to control, and fast in processing. It returns the clusters as image segments. However, the drawback is that multiple small segments may be separated by cutting small sets of isolated nodes in the graph.

Apart from the highlighted methods, the accuracies of the maximum entropy, Isodata, cross entropy, and minimum error thresholding methods are very close to real requirements. There is a high possibility that the accuracy can be further improved by adding more effective pre- and postprocessing steps. However, the fuzzy entropy, adaptive thresholding, and fuzzy c-means clustering methods exhibit very low accuracy. The accuracies of these methods can be improved by combining them with appropriate segmentation methods. In the future, integrated or hybrid segmentation methods will be studied. This study covers only the extraction of the nuclei regions from surrounding objects in the entire image. The examined methods are not capable of splitting the overlapping cells. It should be noted that the touching or overlapping or clustering cells can be isolated into individual ones in further stages. In order to do so, these methods may integrate with the overlapped object splitting techniques such as the watershed methods, contour concavity methods, rule-based splitting methods, bottleneck method, and so on.

## 5. Conclusions

In this paper, we presented twelve individual image segmentation algorithms to extract the cell nuclei from cytology pleural effusion images. Each method includes three stages: preprocessing, segmentation, and postprocessing. The accuracies (DSC) of the five segmentation methods, namely, Otsu's method, k-means clustering, mean shift, Chan–Vese method, and graph cut method, were 94, 94, 95, 94, and 93%, respectively; the average computational times for an image were 1.08, 36.62, 50.18, 330, and 44.03 seconds, respectively. The results meet clinical requirements. Therefore, they can be practically used as a prerequisite step in developing CAD systems. The Chan–Vese method is computationally expensive compared to others. In contrast, Otsu's method is relatively simple and fast. It is our hope that the results and observations will be useful for current and potential future studies on cytology images of pleural effusion. Unfortunately, the examined methods are not capable of separating the overlapping nuclei. In further study, they need to integrate with additional splitting methods to isolate the overlapping nuclei into individual ones. As part of the future work, more state-of-the-art segmentation methods will be explored. In addition, it would be interesting to combine different segmentation methods for improving the segmentation accuracy; however, this requires research efforts beyond the scope of this paper. It would be worthwhile to study the implementation and combination of different algorithms. Our ultimate goal is to implement an effective CAD system for malignant pleural effusion.

## Figures and Tables

**Algorithm 1:**
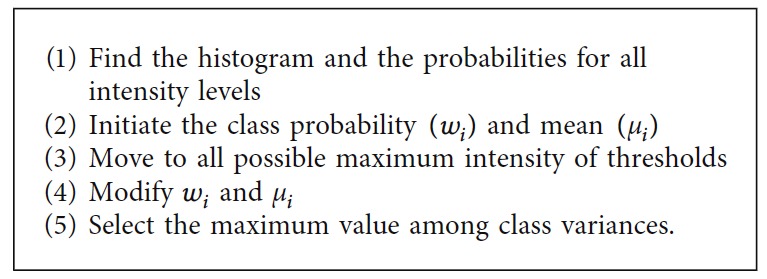
Otsu's thresholding method.

**Algorithm 2:**
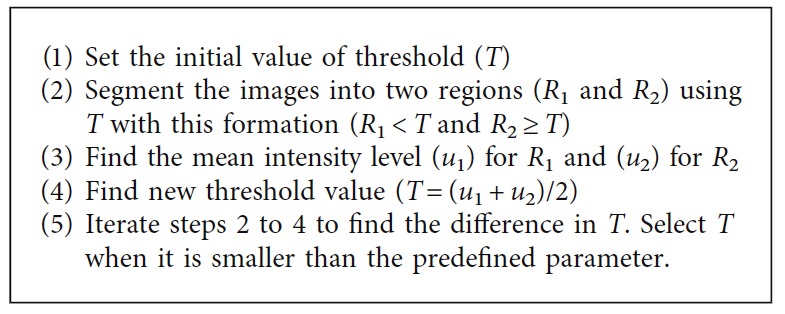
Isodata thresholding method.

**Algorithm 3:**
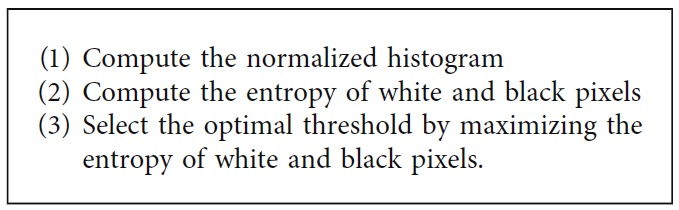
Maximum entropy thresholding method.

**Algorithm 4:**
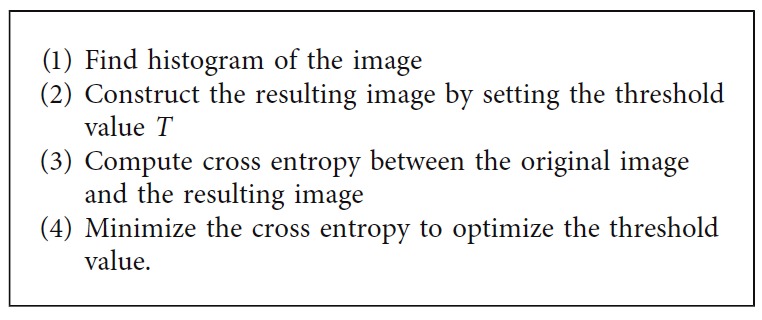
Cross entropy thresholding method.

**Algorithm 5:**
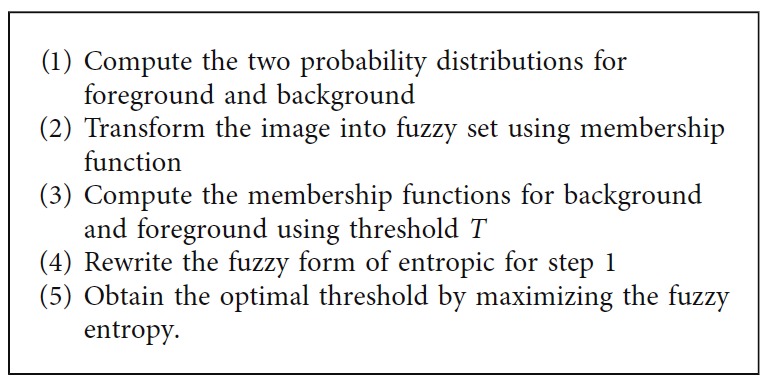
Fuzzy entropy thresholding method.

**Algorithm 6:**
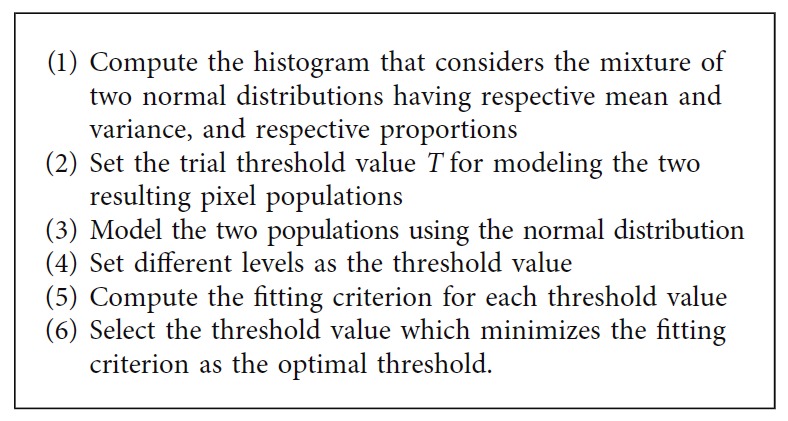
Minimum error thresholding method.

**Algorithm 7:**
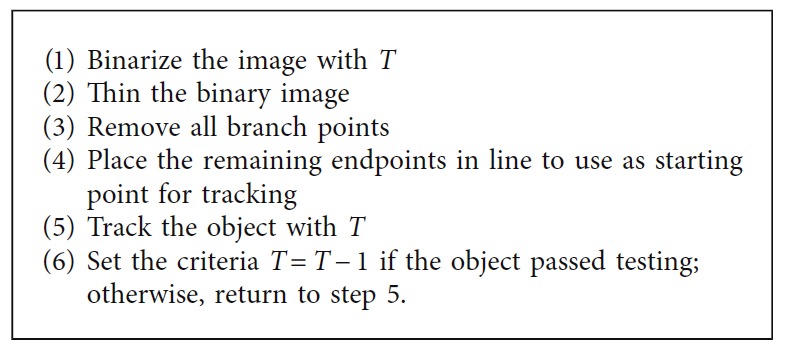
Adaptive thresholding method.

**Algorithm 8:**
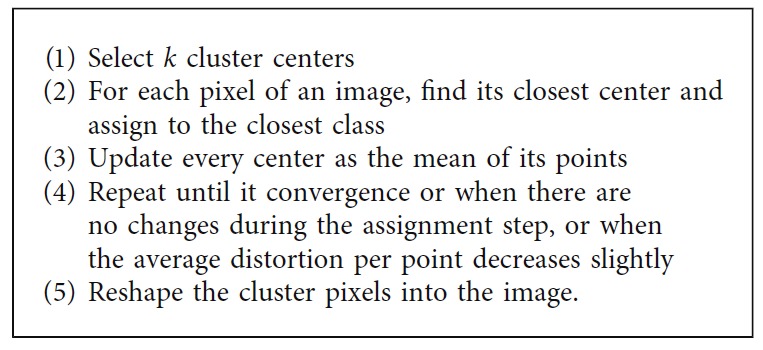
K-means clustering method.

**Algorithm 9:**
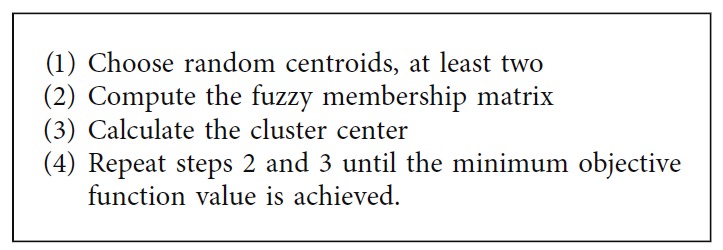
Fuzzy c-means clustering method.

**Algorithm 10:**
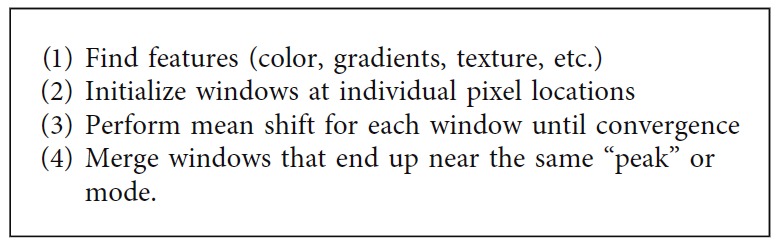
Mean shift clustering method.

**Pseudocode 1:**
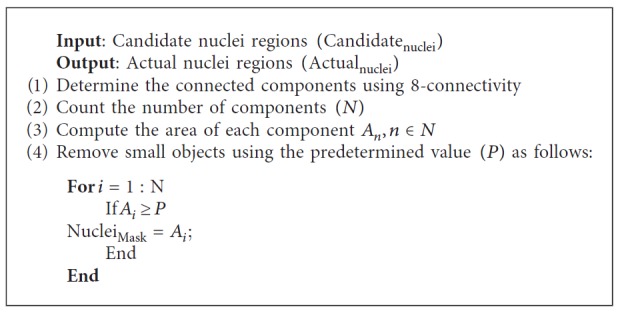
Pseudocode for removing the false findings.

**Table 1:** Quantitative experimental results.

Methods/evaluation	Pre	Re	Fm	JI	DSC (%)
Otsu's method	0.91	0.89	0.90	0.89	94
Isodata	0.86	0.84	0.85	0.84	91
Maximum entropy	0.84	0.94	0.88	0.94	91
Cross entropy	0.94	0.82	0.87	0.82	90
Minimum error	0.85	0.82	0.83	0.82	89
Fuzzy entropy	0.68	0.67	0.68	0.67	80
Adaptive thresholding	0.96	0.50	0.66	0.50	67
k-means	0.90	0.89	0.89	0.89	94
Fuzzy c-means	0.94	0.77	0.85	0.77	77
Mean shift	0.93	0.91	0.92	0.91	95
Chan–Vese method	0.89	0.87	0.88	0.87	94
Graph-based min cut	0.87	0.95	0.91	0.87	93
